# Study on insulin resistance and ischemic cerebrovascular disease: A bibliometric analysis *via* CiteSpace

**DOI:** 10.3389/fpubh.2023.1021378

**Published:** 2023-03-06

**Authors:** Xue Zhou, Chen Kang, YuHong Hu, XingChen Wang

**Affiliations:** ^1^First Clinical Medical College, Shandong University of Traditional Chinese Medicine, Jinan, China; ^2^Division of Neurology, Affiliated Hospital of Shandong University of Traditional Chinese Medicine, Jinan, China; ^3^Division of Cardiology, The 960th Hospital of the PLA Joint Logistic Support Force, Jinan, China; ^4^Department of Neurology, The Second Affiliated Hospital of Shandong University of Traditional Chinese Medicine, Jinan, China; ^5^The Second Clinical Medical College, Shandong University of Traditional Chinese Medicine, Jinan, China

**Keywords:** insulin resistance, ischemic cerebrovascular disease, association, oxidative stress, inflammation, CiteSpace

## Abstract

**Background:**

It is reported that insulin resistance widely exists in non-diabetic patients with a recent history of transient ischemic attack (TIA) or ischemic stroke. There is currently strong evidence to prove the bidirectional effect of glucose metabolism disorders and stroke events. Therefore, it is necessary to retrospectively tease out the current status, hotspots, and frontiers of insulin resistance and ischemic cerebrovascular disease through CiteSpace.

**Materials and methods:**

We searched the Web of Science (WOS) for studies related to insulin resistance and ischemic cerebrovascular disease from 1999 to April 2022, then downloaded the data into CiteSpace to generate a knowledge visualization map.

**Results:**

A total of 1,500 publications relevant to insulin resistance and ischemic cerebrovascular disease were retrieved. The USA had the most articles on this topic, followed by PEOPLES R CHINA and JAPAN. WALTER N KERNAN was the most prolific author, whose research mainly focused on insulin resistance intervention after stroke (IRIS) trial. The most common keywords were myocardial ischemia, metabolic syndrome, ischemic stroke, cerebral ischemia, association, oxidative stress, inflammation, and adipose tissue. Major ongoing research trends include three aspects: (1) the association between insulin resistance and ischemic cerebrovascular disease in non-diabetic patients, (2) the intrinsic pathological mechanism between insulin resistance and ischemic cerebrovascular disease, and (3) early intervention of insulin resistance to improve the prognosis of stroke.

**Conclusion:**

The results of this bibliometric study provide the current status and trends of clinical research publications in the field of insulin resistance and ischemic cerebrovascular disease. Insulin resistance is strongly associated with the occurrence of ischemic stroke, early neurological deterioration in stroke patients, post-stroke depression, and cerebral small vessel disease. Early treatment of insulin resistance can be an effective way to prevent the onset of ischemic stroke and improve stroke prognosis. This study may help researchers to identify hot topics and explore new research directions.

## 1. Introduction

Stroke is the second leading cause of death and the third leading cause of disability worldwide (1). An estimated 6.6 million Americans over the age of 20 have a stroke, occurring on average every 40 s and dying every 4 min (2, 3). In China, the mortality rate of stroke is as high as 149.49 per 100,000 (about 1.57 million deaths), accounting for 22.33% of the total deaths (4). Over the past decade, great advances have been made for cerebrovascular disease, many risk factors such as hypertension, diabetes, atrial fibrillation, smoking, alcohol abuse, obesity, and carotid stenosis have been found (5), and many effective methods have emerged for prevention and treatment, which include controlling blood pressure, blood lipids, blood sugar, anti-thrombotic therapy, smoking cessation, regular physical activity, etc. It does reduce the incidence, recurrence, and mortality rate of stroke events. However, about 790,000 individuals in the United States still have stroke events every year, with ischemic stroke accounting for 87% of all stroke events. Projections show that by 2030, an additional 3.4 million people aged ≥18 years will have had a stroke, a 20.5% increase in prevalence from 2012 (2, 3). This reminds us that some unrecognized and unappreciated vascular risk factors still need to be intervened early.

Insulin resistance is clinically defined as a decrease in glucose uptake and utilization capacity by exogenous or endogenous insulin when compared to the normal population (6). There is currently strong evidence to prove the bidirectional effect of glucose metabolism disorders and stroke events. Insulin resistance can be used as an early predictor and independent risk factor of cardiovascular and cerebrovascular events (7, 8), and stroke can aggravate glucose metabolism disorders and lead to insulin resistance (9). Some researchers have observed that rats with middle cerebral artery occlusion show decreased insulin secretory capacity and insulin sensitivity after 1 day of cerebral ischemia, accompanied by elevated fasting glucose and fasting insulin levels (10). The American Heart Association/American Stroke Association guidelines for the Prevention of Stroke in Patients with Stroke and Transient Ischemic Attack (TIA) published in 2021 state that ~ 30% of ischemic stroke patients are pre-diabetic and 50% of non-diabetic ischemic stroke patients have insulin resistance (11–13). Another study has shown that impaired insulin sensitivity is very prevalent in non-diabetic patients with a recent TIA or non-disabling ischemic stroke (12). Such abnormalities in glycemic traits persist even after the stressful state has disappeared and can exacerbate neurological damage, leading to a poor prognosis. This reminds us that combing the relationship between insulin resistance and ischemic cerebrovascular disease can provide more therapeutic approaches for early intervention and reduce the incidence of stroke, as well as post-stroke adverse events. Therefore, it is necessary to analyze the current status, hotspots, and frontiers of insulin resistance and ischemic cerebrovascular disease visually by using CiteSpace (14).

CiteSpace is an information visualization software developed by Professor Chaomei Chen using Java language (15, 16), which is mainly used to explore the frontiers of discipline development and research status. This study included the literature related to insulin resistance and ischemic cerebrovascular disease, and analyze the research direction and hotspots knowledge by using CiteSpace software.

## 2. Materials and methods

### 2. 1. Search strategy

We searched for relevant studies using the following terms: (Lacunar infarction^*^) OR (Ischemic cerebrovascular disease^*^) OR (Ischemic^*^) OR (Brain Ischemia^*^) OR (Ischemic Encephalopathy^*^) OR (Cerebral Ischemia^*^) OR (Ischemic Stroke^*^) OR (Cryptogenic Stroke^*^) OR TIA OR (Cerebral Infarctions^*^) OR (Subcortical Infarction^*^) OR Transient Ischemic Attack and (Insulin resistance^*^). The type of literature is limited to “article” or “review,” the language is limited to English, and the citation index is selected from the Web of Science Core Collection for the period 1999 to April 2022. Finally, a total of 1,905 articles were obtained.

### 2.2. Analysis tool–CiteSpace

Set up the CiteSpace folder with four subfolders: input, output, data, and project. The complete records and references of 1,905 articles retrieved from WOS were exported in plain text format, named “download_XX.txt,” and then imported into the input folder for analysis in CiteSpace 5.8 R3 software. After eliminating the duplicate literature, 1,500 articles were left. Time slicing was performed from January 1999 to April 2022, years per slice was set to one, selection criteria top N was set to 50, pruning selected pathfinder, pruning sliced networks simplified atlas, node types selected author, institution, country, keywords, and reference.

The output results mainly include the annual publication analysis, countries and institutions cooperation analysis, co-authors analysis, co-occurring keywords and cluster analysis, keywords with citation bursts, and co-cited reference analysis. Relevant contents are visualized as knowledge maps, and key nodes and links in different maps are interpreted and analyzed (17). The node represents the analyzed research object. The higher the frequency of occurrence, the larger the range of nodes. The color and thickness of the node inner circle represent the frequency of occurrence in different periods. If the node has a purple outer circle, it represents a high betweenness centrality. Centrality reflects the role of the nodes in the knowledge network, nodes with high centrality (>0.1) were usually considered turning points or pivotal points in a field (15, 16). The links between the nodes represent the co-occurrence relationship, and the thickness of the links represents the strength of the co-occurrence. The more the links, the thicker, and the closer relationship between the nodes. CiteSpace provides module value (Q value) and average silhouette value (silhouette, S value) based on the network structure and clustering results. It is generally believed that a Q value > 0.3 (empirical value) means that the cluster structure is significant, an S value at 0.7 clusterings has certain significance and credibility, and above 0.5 that clustering is generally reasonable. The larger the cluster structure, the smaller the cluster ID (18).

## 3. Results

### 3.1. Annual publications analysis

**Figure 1:**
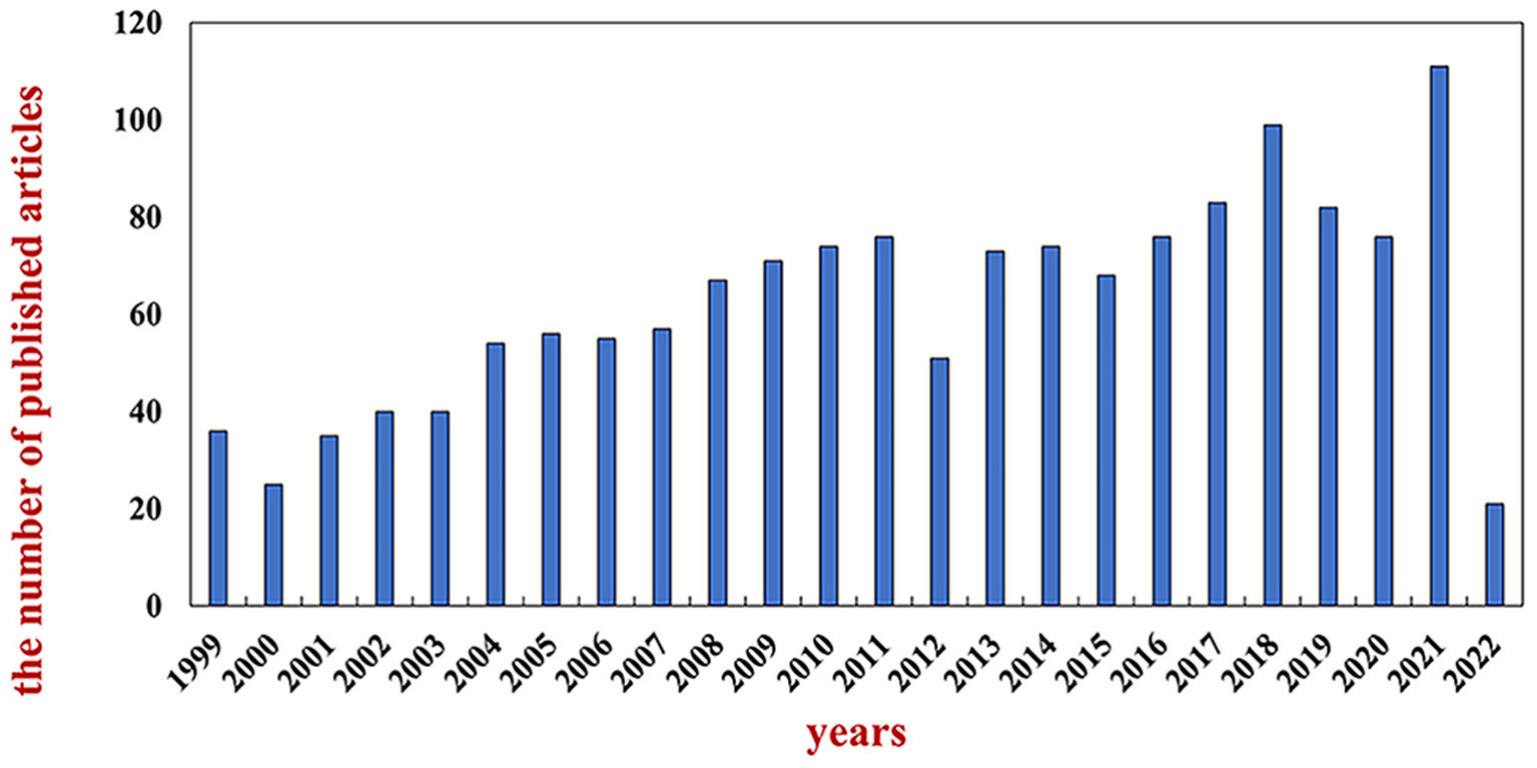
Annual trend chart of publications.

In this study, 1,500 included documents (publications in 2022 were not fully included) were statistically analyzed according to the published time, and the annual publication trends are shown in Figure 1. The overall number of documents showed a fluctuating upward trend. In the initial stage, the publication volume increased slowly from 1999 to 2011; from 2011 to 2015, there were temporary fluctuations, with a slight continued upward trend in the overall amount of publications; from 2015 to 2021, the growth rate of publications increased significantly and at a faster pace, reaching two peaks in 2018 and 2021.

### 3.2. Countries and institutions analysis

**Figure 2:**
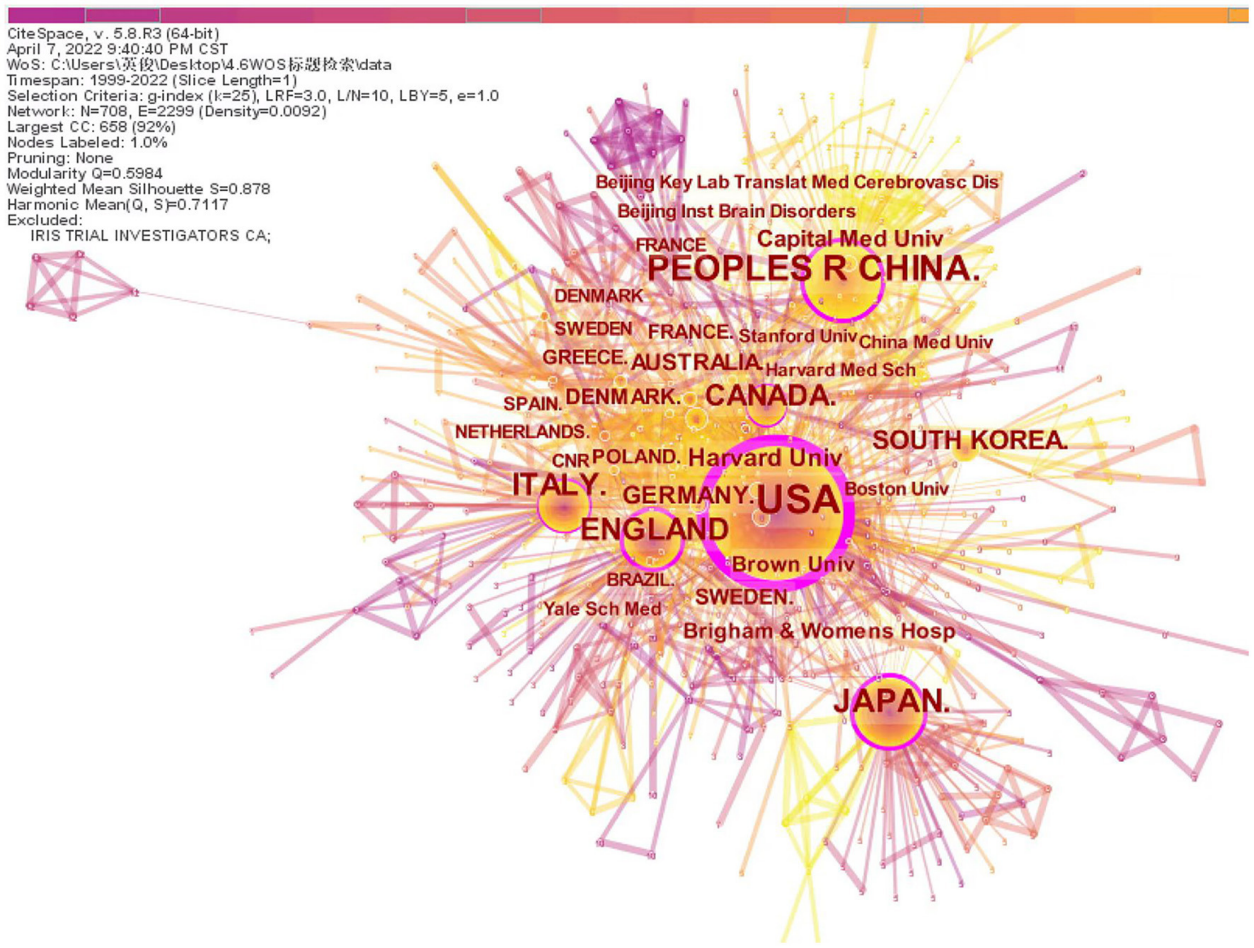
The network of countries and institutions.

Generate co-occurrence network analysis of countries and institutions (Figure 2). Node *N* = 708, link E = 2,299, module value Q = 0.5984, S value = 0.878. Each node represents a country or institution. The top 10 countries are the United States, China, Japan, England, Italy, Canada, South Korea, Germany, Australia, and France, and those with high centrality (>0.10) are the United States, China, England, Italy, Germany, and Australia, which indicate that these countries have a certain influence in the study of the correlation between insulin resistance and ischemic cerebrovascular disease. The top three countries contributed 850 articles, accounting for 56.6% of all the included documents. The top five institutions are mainly universities, including Harvard University, Capital Medical University, Brown University, Brigham and Women's Hospital, and Boston University (Table 1). There is active cooperation between various countries and institutions, especially in the USA, China, and Japan.

**Table 1:** Top 10 countries and top five institutes publishing research on insulin resistance and ischemic cerebrovascular disease.

**Rank**	**Country/ region**	***N***	**Centrality**	**Institution**	***N***	**Centrality**
1	USA	470	0.39	Harvard University	34	0.09
2	Peoples R China	251	0.11	Capital Med University	32	0.03
3	Japan	139	0.03	Brown University	24	0.06
4	England	109	0.17	Brigham and Womens Hospital	23	0.02
5	Italy	107	0.16	Boston University	16	0.05
6	Canada	84	0.09			
7	South Korea	61	0.00			
8	Germany	51	0.13			
9	Australia	42	0.11			
10	France	40	0.02			

### 3.3. Co-authors analysis

**Figure 3:**
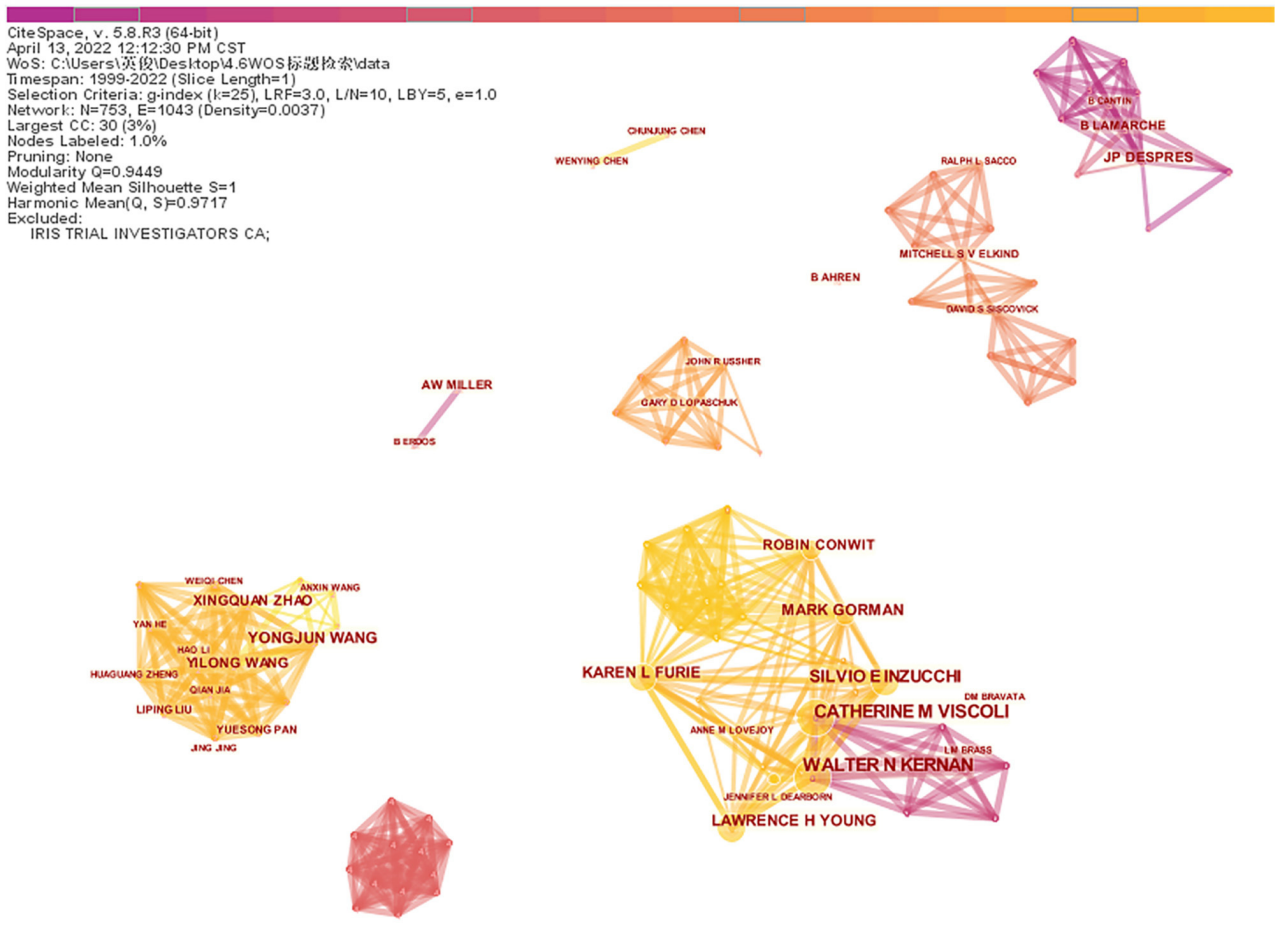
The network of co-authors.

Generate co-occurrence network analysis of authors (Figure 3). The results show the number of nodes *N* = 753, link E = 1,043, Q = 0.9449, S = 1, representing a total of 753 authors included and 1,043 collaborations between authors. The top 10 authors can be grouped into two main categories (Table 2). The first category is a collaboration cluster diagram consisting of WALTER N KERNAN (21 articles), CATHERINE M VISCOLI (18 articles), SILVIO E INZUCCHI (13 articles), KAREN L FURIE (12 articles), LAWRENCE H YOUNG (11 articles), MARK GORMAN (10 articles), and ROBIN CONWIT (eight articles). The second category is the cooperative cluster diagram composed of YONGJUN WANG (10 articles), XINGQUAN ZHAO (eight articles), and YILONG WANG (seven articles).

**Table 2:** Top 10 authors with the most publications on insulin resistance and ischemic cerebrovascular disease.

**Rank**	**Author**	***N***	**Year**
1	Walter N. Kernan	21	2003
2	Catherine M. Viscoli	18	2002
3	Silvio E. Inzucchi	13	2016
4	Karen L. Furie	12	2016
5	Lawrence H. Young	11	2017
6	Yongjun Wang	10	2017
7	Mark Gorman	10	2016
8	Robin Conwit	8	2017
9	Xingquan Zhao	8	2016
10	Yilong Wang	7	2017

In the first cooperative relationship cluster map, the author with the most publications was WALTER N KERNAN (21 articles). From 2003 to 2005, WN KERNAN's research team recognized the association between insulin resistance and increased risk of ischemic vascular events, that decreased insulin sensitivity and impaired glucose tolerance widely existed in patients with a recent history of TIA or ischemic stroke, using pioglitazone intervention can improve post-stroke insulin resistance (19–21). Subsequently, they initiated an international, multicenter, randomized, double-blind, placebo-controlled study IRIS trial (22). The results were published from 2016 to 2019, demonstrating that treatment with pioglitazone improves cardiovascular outcomes of non-diabetic, insulin-resistant patients with stroke or TIA (23). In the second cooperative relationship cluster map, the most published author was YONGJUN WANG (10 articles). YJ Wang's study began in 2017, to investigate the impact of insulin resistance on the prognosis of non-diabetic ischemic stroke patients through cross-sectional studies and cohort studies based on the Chinese population (24).

### 3.4. Co-cited references analysis

**Figure 4:**
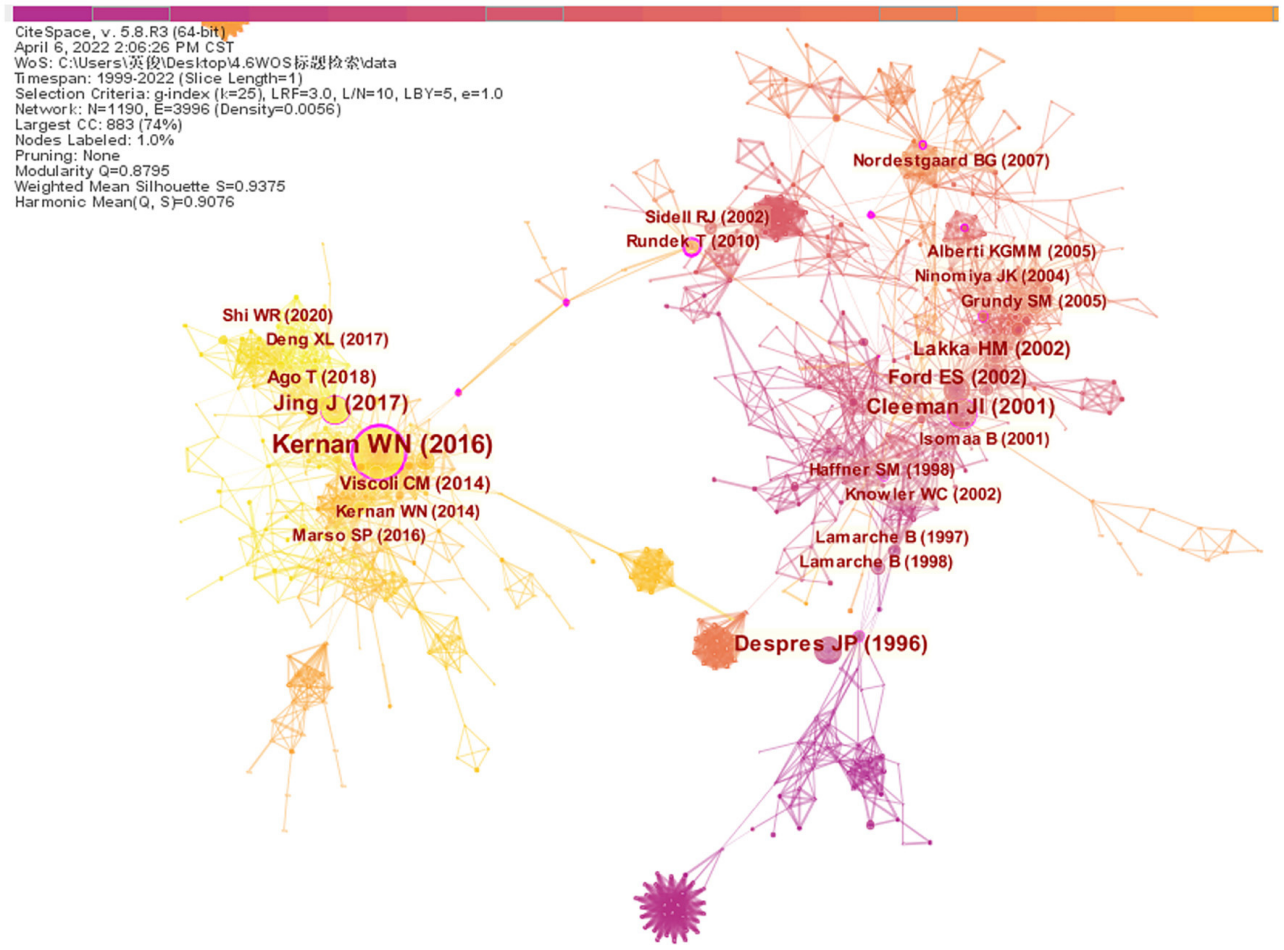
The network of co-cited references.

Generate co-cited references analysis network map (Figure 4). The results show the number of nodes *N* = 1,190, link E = 3,996, Q = 0.8795, and S = 0.9375. Each node represents a co-cited reference, the larger the node, the higher the citation frequency, and the links represent the co-citation relationship between different articles. The purple outer circle of the node represents that the references have high betweenness centrality, which is of great research significance in the development process of this field (25).

The top 10 co-cited references ranked according to frequency are listed in Table 3, and they were co-cited more than 200 times. Most of the articles involved are original research papers, laying the knowledge basis for further research on insulin resistance and ischemic cerebrovascular disease. The first high-frequency co-cited reference was the article published in the New Engl J MED by WN Kernan et al. (23), with high centrality, which demonstrated that the application of pioglitazone can benefit patients without diabetes who had insulin resistance along with a recent history of ischemic stroke or TIA through a multicenter, double-blind and randomized controlled trial study of 3,876 patients with ischemic stroke or TIA. The third co-citation article (26), published in 1996, identified fasting insulin concentration as an independent risk factor for ischemic heart disease through a prospective cohort study of Canadian men. The fourth co-cited reference was published on STROKE by Jing et al. (24), which investigated the relationship between insulin resistance and outcome in non-diabetic patients with first-ever acute ischemic stroke through a cross-sectional survey of 1,245 Chinese, and concluded that insulin resistance was associated with an increased risk of death, stroke recurrence, and adverse outcome but not dependence in non-diabetic patients with acute ischemic stroke. The eighth co-citation reference (27) demonstrated that insulin resistance was independently associated with poor functional outcomes in patients with acute ischemic stroke, apart from the risk of short-term stroke recurrence or death.

**Table 3:** Top 10 co-cited references with the highest frequency on insulin resistance and ischemic cerebrovascular disease.

**Rank**	**Co-cited reference**	**Impact factor**	**Frequency**	**Centrality**
1	Kernan WN, 2016, NEW ENGL J MED, V374, P1321, doi: 10.1056/NEJMoa1506930	91.245	60	0.42
2	Cleeman JI, 2001, JAMA-J AM MED ASSOC, V285, P2486, doi: 10.1001/jama.285.19.2486	56.272	28	0.15
3	Despres JP, 1996, NEW ENGL J MED, V334, P952, doi: 10.1056/NEJM199604113341504	91.245	28	0.00
4	Jing J, 2017, STROKE, V48, P887, doi: 10.1161/STROKEAHA.116.015613	9.910	26	0.13
5	Ford ES, 2002, JAMA-J AM MED ASSOC, V287, P356, doi: 10.1001/jama.287.3.356	7.914	24	0.07
6	Lakka HM, 2002, JAMA-J AM MED ASSOC, V288, P2709, doi: 10.1001/jama.288.21.2709	7.914	21	0.03
7	Viscoli CM, 2014, AM HEART J, V168, P823, doi: 10.1016/j.ahj.2014.07.016	4.749	17	0.01
8	Ago T, 2018, NEUROLOGY, V90, P0, doi: 10.1212/WNL.0000000000005358	9.910	16	0.05
9	Isomaa B, 2001, DIABETES CARE, V24, P683, doi: 10.2337/diacare.24.4.683	19.112	14	0
10	Grundy SM, 2005, CIRCULATION, V112, P2735, doi: 10.1161/CIRCULATIONAHA.105.169404	29.690	14	0.01

The top 10 co-cited references ranked according to betweenness centrality are listed in Table 4, serving as a turning point and bridge for the key nodes of this study. The article types mainly include original research papers and reviews. The first co-cited reference (28) with the highest centrality through a cohort study of 1,509 non-diabetic patients in North Manhattan, found that insulin resistance could be used as a predictor of increased risk of incident stroke in non-diabetic individuals. The second co-cited reference (29) used acute ischemic brain injury rat models induced by ligating the right middle cerebral artery and bilateral common carotid arteries, to examine the relationship between adipocytokines and poststroke hyperglycemia. It was found that stroke rats developed glucose intolerance on days 1 and 2 after cerebral ischemic injury, and fasting blood insulin levels and insulin resistance index were higher in stroke rats than in the sham group. Eventually, it proved that sympathetic system excitation after cerebral ischemia and inducing the secretion of proinflammatory cytokines (TNF-α and MCP-1) from adipose tissue may be an underlying mechanism for the disorder of glucose metabolism in rats. The fourth co-cited reference (30) using meta-analysis concluded that rosiglitazone treatment for type 2 diabetes may increase the incidence of myocardial infarction and cardiovascular adverse events. The fifth co-cited reference (31) proved that applying pioglitazone as agonists of peroxisome proliferator-activated receptor gamma (PPAR gamma) can reduce the incidence of macrovascular events in patients with type 2 diabetes. The sixth co-cited reference (32), as part of the North Manhattan study, identified metabolic syndrome as an important risk factor for ischemic stroke through a prospective cohort study. The eighth co-cited reference (33) found an increased risk of progressive carotid atherosclerosis and coronary heart disease in subjects with metabolic syndrome.

**Table 4:** Top 10 co-cited references with the highest centrality on insulin resistance and ischemic cerebrovascular disease.

**Rank**	**Co-cited reference**	**Impact Factor**	**Centrality**	**Frequency**
1	Rundek T, 2010, ARCH NEUROL-CHICAGO, V67, P1195, doi: 10.1001/archneurol.2010.235		0.53	14
2	Wang YY, 2011, AM J PHYSIOL-ENDOC M, V300, P0, doi: 10.1152/ajpendo.00301.2010	4.310	0.5	6
3	Kernan WN, 2016, NEW ENGL J MED, V374, P1321, doi: 10.1056/NEJMoa1506930	91.245	0.42	60
4	Nissen SE, 2007, NEW ENGL J MED, V356, P2457, doi: 10.1056/NEJMoa072761	91.245	0.34	7
5	Dormandy JA, 2005, LANCET, V366, P1279, doi: 10.1016/S0140-6736(05)67528-9	79.321	0.29	10
6	Boden-Albala B, 2008, STROKE, V39,P30, doi: 10.1161/STROKEAHA.107.496588	9.910	0.19	8
7	Haffner SM, 1998, NEW ENGL J MED, V339, P229, doi: 10.1056/NEJM199807233390404	91.245	0.16	13
8	Bonora E, 2003, DIABETES CARE, V26, P1251, doi: 10.2337/diacare.26.4.1251	19.112	0.16	8
9	Cleeman JI, 2001, JAMA-J AM MED ASSOC, V285, P2486, doi: 10.1001/jama.285.19.2486	56.272	0.15	28
10	Jing J, 2017, STROKE, V48, P887, doi: 10.1161/STROKEAHA.116.015613	9.910	0.13	26

### 3.5. Co-occurring keywords and cluster analysis

**Figure 5:**
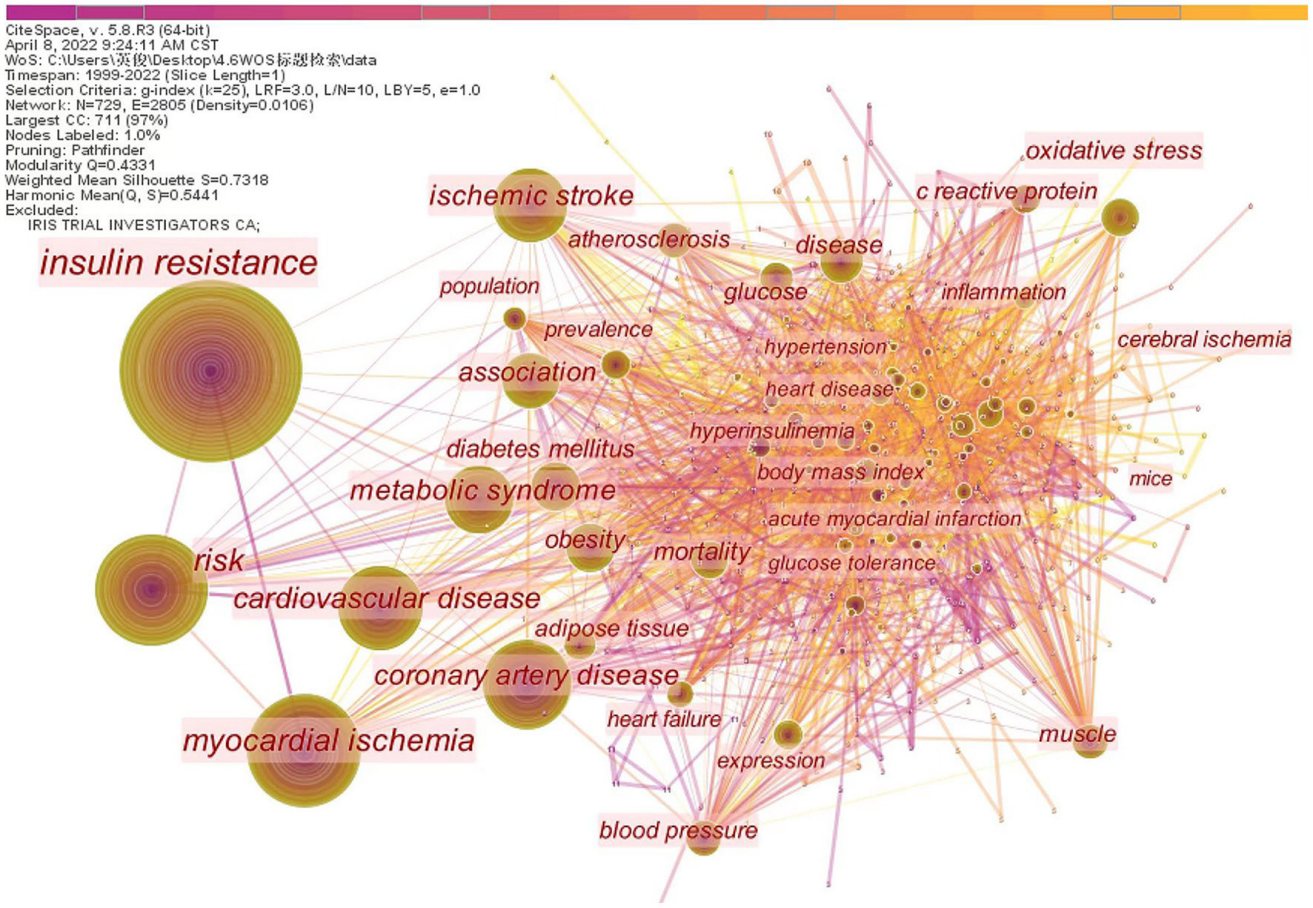
The network of co-occurring keywords.

Generate co-occurring keywords analysis network map (Figure 5). Results showed that node *N* = 729, link E = 2805, Q = 0.4331, S = 0.7318. A total of 729 keywords were included, with a high degree of correlation between them. Keywords can represent the hotspot and trend of research (34). As shown in Table 5, hotspot keywords are listed based on the frequency of occurrence and mediation centrality (>0.1). Keywords of diseases related to insulin resistance mainly include myocardial ischemia, cardiovascular disease, coronary artery disease, metabolic syndrome, ischemic stroke, diabetes mellitus, hypertension, cerebral ischemia et al. The hotspot keywords of the research direction are association, risk, and prevalence, and the mechanism involved is mainly oxidative stress and inflammation. Among them, adipose tissue, blood pressure, and coronary artery disease have better centrality.

**Table 5:** Top 10 keywords in frequency and top 5 keywords in centrality on insulin resistance and ischemic cerebrovascular disease.

**Rank**	**Frequency**	**Keywords**	**Centrality**	**Keywords**
1	930	Insulin resistance	0.11	Coronary artery disease
2	379	Risk	0.11	Disease
3	369	Myocardial ischemia	0.11	Blood pressure
4	251	Cardiovascular disease	0.10	Muscle
5	243	Coronary artery disease	0.10	Adipose tissue
6	211	Ischemic stroke		
7	184	Metabolic syndrome		
8	136	Association		
9	126	Diabetes mellitus		
10	114	Oxidative stress		

**Figure 6:**
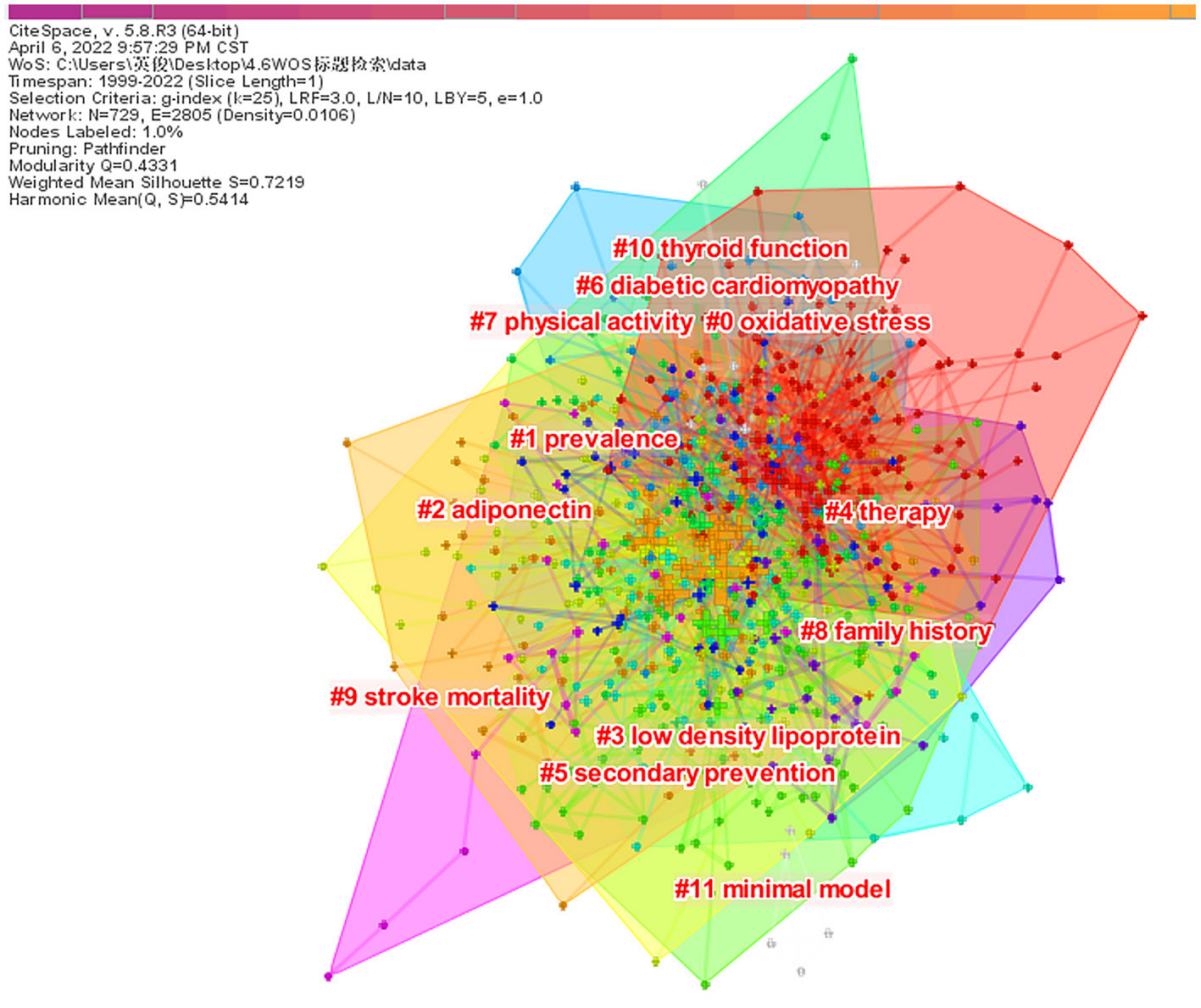
Keywords cluster analysis co-occurrence map.

Clustering analysis of co-occurrence keywords was performed, and cluster labels revealed major themes in the research field (Figure 6). We eventually obtained 11 clusters, each with a silhouette value above 0.6, indicating that the clustering results are reliable and meaningful. The cluster label keywords were extracted: #0 oxidative stress, #1 prevalence, #2 adiponectin, #3 low density lipoprotein, #4 therapy, #5 secondary prevention, #6 diabetic cardiomyopathy, #7 physical activity, #8 family history, #9 stroke mortality, #10 thyroid function, and #11 minimal model (Table 6).

**Table 6:** Keywords cluster analysis.

**Cluster-ID**	**Size**	**Silhouette**	**Coverage**	**Label**
0	142	0.616	Oxidative stress; gene expression; cerebral ischemia; nitric oxide synthase; Alzheimer's disease	Oxidative stress
1	111	0.736	Prevalence; hyperinsulinemia; cardiovascular disease; population; risk factor	Prevalence
2	82	0.715	Adiponectin; resistin; ischemic stroke; inflammation; c reactive protein	Adiponectin
3	80	0.787	Low density lipoprotein; cardiovascular risk factor; coronary artery disease; von willebrand factor; risk	Low density lipoprotein
4	78	0.627	Therapy; hyperglycemia; stroke severity; interleukin-1; tissue plasminogen activator	Therapy
5	56	0.799	Secondary prevention; pioglitazone; clinical trial; transient ischemic attack; obesity	Secondary prevention
6	46	0.791	Diabetic cardiomyopathy; heart failure; trimetazidine; fatty acid oxidation; cardiomyopathy	Diabetic cardiomyopathy
7	44	0.774	Physical activity; randomized controlled trial; cardiac rehabilitation; fatty acid; neuroscience	Physical activity
8	31	0.877	Family history; hemostasis; factor alpha; thrombosis; plasminogen activator inhibitor 1 (pai-1)	Family history
9	25	0.899	Stroke mortality; transplantation; renal failure; kidney; cardiovascular disease (CVD)	Stroke mortality
10	7	0.987	Thyroid function; turner syndrome; PKC delta; puberty; genes	Thyroid function

### 3.6. Keywords with citation bursts

**Figure 7:**
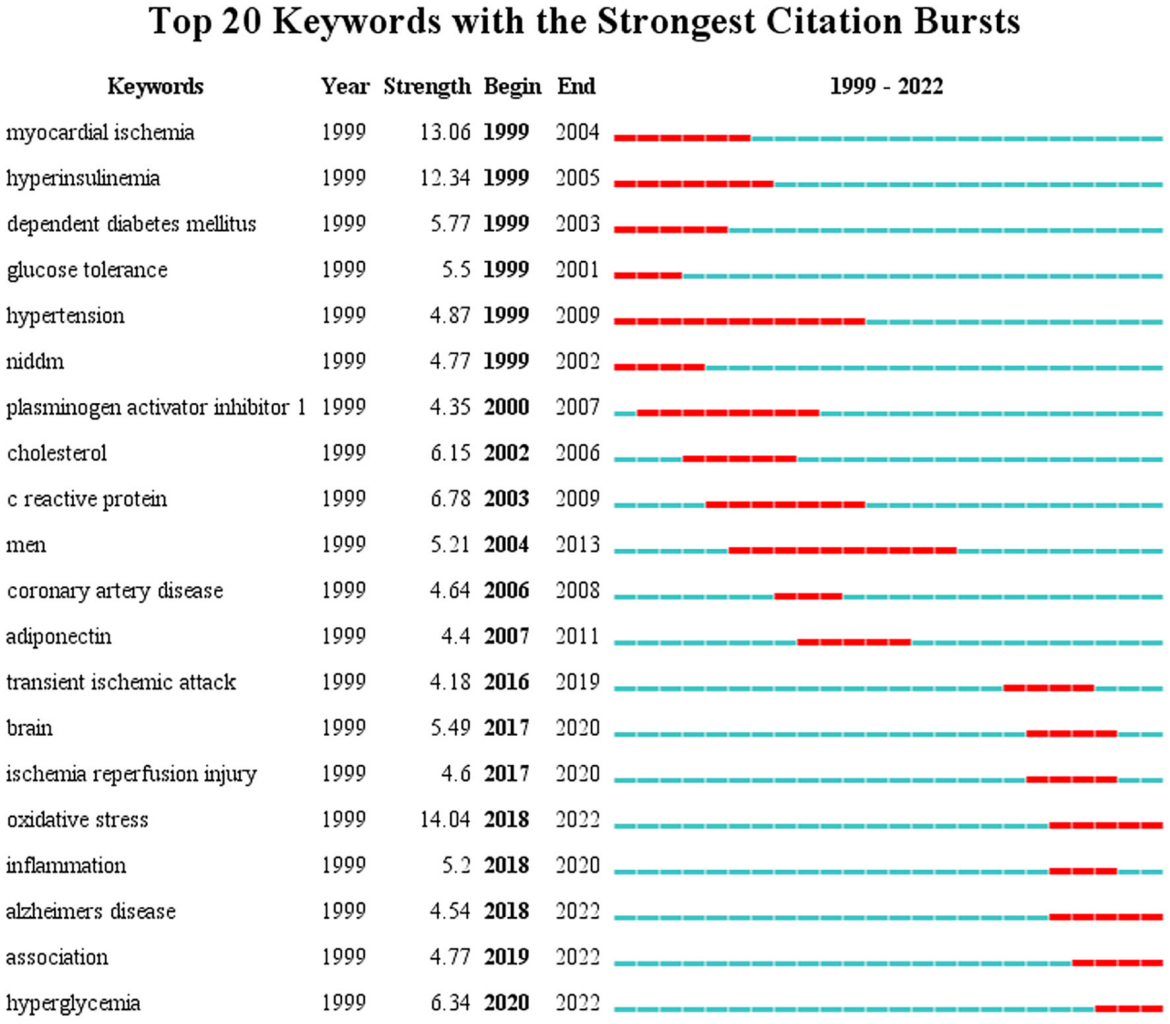
Top 20 keywords with the strongest citation bursts.

The top 20 keywords with the strongest citation burst from 1999 to 2022 are shown in Figure 7. The blue line indicates the time interval, and the red line indicates the period when the keyword burst occurs. “Burst words” refer to words that are frequently cited over a period of time (35). It can predict the migration and changes in research frontiers according to the distribution of keywords with the strongest citation burst. In the previous period, the relationship between glucose metabolism disorders and ischemic cardiomyopathy, hypertension, and coronary artery disease was mainly studied; from 2000 to 2011, research focused on the mechanisms of intrinsic associations between diseases, including plasminogen activator inhibbitor1, adiponectin, cholesterol, and c reactive protein; in the past 6 years, more attention has been paid to the association between insulin resistance and TIA, ischemia-reperfusion injury and Alzheimer's disease. The pathological mechanisms have focused on oxidative stress and inflammation, and more emphasis is placed on the relationship between insulin resistance and other diseases. The emerging research keywords in recent years represent the current research trends and hotspots in this field.

## 4. Discussion

### 4.1. Research progress in insulin resistance and ischemic cerebrovascular disease

Our results show that from 1999 to 2022, a growing number of studies confirm the strong link between insulin resistance and ischemic cerebrovascular disease, indicating that the field has attracted lively discussion and widespread concern in recent years. The United States, China, and Japan are the largest contributors to the number of publications and have played an important role in the development of the field. However, there are not enough collaborative relationships between authors from different countries and regions, and there is an urgent need to strengthen cooperation between countries, institutions, and authors to conduct relevant research. Major ongoing research trends include three aspects: (1) the association between insulin resistance and ischemic cerebrovascular disease in non-diabetic patients, (2) the intrinsic pathological mechanism between insulin resistance and ischemic cerebrovascular disease, and (3) early intervention of insulin resistance to improve the prognosis of stroke.

### 4.2. Hot issue of insulin resistance and ischemic cerebrovascular disease

#### 4.2.1. Association

The association between insulin resistance and ischemic cerebrovascular disease has received hot attention in recent years. In clinical studies of insulin resistance, evaluation indexes are more mixed, mostly assessed by Homeostasis Model Assessment-Insulin Resistance (HOMA-IR) index (36), or triglyceride-glucose(Ty-G) index (37).

##### 4.2.1.1. Insulin resistance and silent lacunar infarction

Two studies from Korea (38) and Japan (39) evaluated the association between insulin resistance and silent lacunar infarction (SLI), and found that insulin resistance was an independent risk factor for SLI and was positively associated with the incidence and severity of SLI. In elderly patients, impaired insulin sensitivity and decreased muscle strength jointly increased the high risk of SLI.

##### 4.2.1.2. Insulin resistance and cerebral small vessel disease

A Japanese study (40) evaluated the association between insulin resistance and cerebral white matter lesions in non-diabetic patients with ischemic stroke. Insulin resistance was defined as HOMA-IR index ≥ 2.5, the degree of periventricular hyperintensity (PVH), as well as deep and subcortical white matter hyperintensity (DSWMH) were measured using brain MRI. The results revealed that insulin resistance was closely related to cerebral white matter lesions in non-diabetic patients with non-cardiac ischemic stroke, with higher HOMA-IR index in patients with heavier PVH and DSWMH. Another study prospectively recruited older, non-diabetic, healthy subjects to assess the association between insulin resistance and the overall cerebral small vessel disease (CSVD) burden (41). The HOMA-IR index ≥ 2.80 was defined as Insulin resistance. The results of the study ultimately showed that Insulin resistance, independent of other clinical risk factors, is positively associated with increased severity of overall CSVD burden in a dose-dependent manner.

##### 4.2.1.3. Insulin resistance and post-stroke depression

Two prospective cohort studies in China demonstrated that prediabetes (impaired fasting glucose, impaired glucose tolerance, or HbA1c 5.7–6.4%) was associated with post-stroke depression and could serve as an early predictor (42). In addition, insulin resistance estimated by the HOMA-IR index may have potential clinical significance in identifying stroke patients at risk of developing depression, independent of recognized predictive factors (43).

##### 4.2.1.4. Insulin resistance and risk stratification of ischemic stroke

Triglyceride-glucose index (Ty-G) is considered to be a simple and reliable practical surrogate indicator of insulin resistance. A cross-sectional study from rural areas of northeast China (44) aimed to explore the relationship between Ty-G and ischemic stroke. When Ty-G was divided into quartiles, the risk of ischemic stroke in the top quartile was 1.776 times higher than in the bottom category. Eventually, this finding demonstrates the potential value of Ty-G index in optimizing ischemic stroke risk stratification in the general population. Another retrospective observational multicenter study on the eICU database investigated the prognostic value of the Ty-G index in patients with critically ill stroke (45). The results showed that Ty-G was associated with increased in-hospital mortality in patients with severe ischemic stroke, but not in patients with hemorrhagic stroke. It is therefore concluded that Ty-G may be a potential predictor of hospital and ICU mortality in critically ill stroke patients, especially in ischemic stroke patients. A study in Jiangxi, China, proved that the Ty-G index is potentially useful in the early identification of elderly hypertensive patients at high risk of experiencing a first stroke (46). A recent meta-analysis of Ty-G and stroke risk (47), including 11 cohort studies, found that the risk of ischemic stroke was positively associated with the Ty-G index, and the elevated Ty-G index was an independent risk factor for stroke occurrence, particularly in ischemic stroke.

##### 4.2.1.5. Insulin resistance and the prognosis of ischemic stroke

Two studies from Yongjun Wang's team used the HOMA-IR index and the Ty-G index representing insulin resistance to determine whether it was associated with an adverse clinical outcome of ischemic stroke. One study (48) proved that the Ty-G index was associated with an increased risk of stroke recurrence, all-cause mortality, and neurological deterioration in patients with ischemic stroke. Another explored relationship between insulin resistance and the risk of early neurological deterioration (END) in patients with non-diabetic acute ischemic stroke (49). Ultimately this study provides strong evidence that insulin resistance may be an independent risk factor for END in non-diabetic patients with acute ischemic stroke. However, whether insulin resistance can be used as an independent risk factor for poor prognosis within urgent 3 months remains controversial. Research from Fukuoka, Japan enrolled 4,655 patients with acute ischemic stroke, finding that HOMA-IR was not associated with stroke recurrence or mortality within 3 months of onset (27).

##### 4.2.1.6. Insulin resistance and atherosclerosis

Metabolic syndrome(MetS) is a group of risk factors associated with insulin resistance (50) and is present in about half of the patients with symptomatic intracranial atherosclerotic stenosis (51). The components of MetS interact to affect vascular thickness synergistically and promote the development of subclinical atherosclerosis (52). Atherosclerosis is the main putative mechanism between insulin resistance and ischemic stroke. One study enrolled 1,523 ischemic stroke patients with Ty-G index and carotid artery imaging data (53). Carotid atherosclerosis was measured by common carotid artery intima-media thickness (cIMT). The result demonstrates that a higher Ty-G index was associated with carotid atherosclerosis in patients with ischemic stroke, suggesting that Ty-G could be a promising atherosclerotic marker.

#### 4.2.2. Oxidative stress and inflammation

Since 2018, more attention has been paid to the pathological processes of oxidative stress and inflammatory response between insulin resistance and ischemic cerebrovascular disease.

When insulin binds to insulin receptors on the plasma membrane, the insulin signaling pathway is activated (54), promoting glucose transport and utilization. In the pathological stage of insulin resistance, glucose utilization and uptake by skeletal muscle, adipose tissue, liver, and other organs are weakened, and the liver function of inhibiting gluconeogenesis and promoting glycogen synthesis is decreased, resulting in an increase in peripheral glucose in the blood. Defective inhibition of lipolysis by insulin leads to increased levels of triglycerides (TG), free fatty acids (FFA), and low-density lipoprotein (LDL), and decreased levels of high-density lipoprotein (HDL) (55). Increased level of FFA induce ROS generation through activation of NADPH oxidation and increased ROS induce oxidative stress and ER stress, stimulating adipose tissue to synthesize and secrete a large number of biologically active substances, such as proinflammatory cytokines, acute phase reactants, angiotensin II, leptin, resistin, adiponectin, and plasminogen activator inhibitor-1(PAI-1) (56). Adipose tissue activates the canonical proinflammatory NF-κB pathway, resulting in increased expression of several proinflammatory cytokines, including NLRP3, TNF-α, IL-1β, IL-6, and MCP-1 (57). All the mechanisms contribute to endothelial dysfunction, atherosclerosis, and thrombosis, leading to cardiovascular and cerebrovascular disease.

The brain has been identified as an insulin-sensitive organ with widely distributed insulin receptors (58). Hyperinsulinemia resulting from systemic insulin resistance causes insulin resistance in central neurons (59), which in turn may exacerbate ischemic cerebrovascular damage by downregulating the PI3K-AKT signaling pathway and inhibiting cell survival. Ischemic injury induces cell death through proapoptotic signaling molecules, including forkhead transcription factor (FKHR), GSK-3β, and Bad, which can be prevented by AKT phosphorylation (60). Insulin and insulin-like growth factor 1 (IGF-1) exert neuroprotective effects by activating PI3K-AKT signaling and preventing decreased AKT phosphorylation during ischemia (61). *In vitro* experiments (62) by simulating insulin resistance models of central cortical neurons, found that long-term exposure to insulin increased Akt activation and severely attenuated this response after subsequent short-term insulin treatment, resulting in reduced neuroprotective effects of insulin and IGF-1. This study demonstrated that central insulin resistance exacerbates cerebral ischemic injury by blunting Akt kinase phosphorylation.

Measures taken against the assembly and activity of the NLRP3 (nucleotide-binding oligomerization domain-like receptor family pyrin domain-containing 3) inflammasome may be a potential and novel therapy for cerebrovascular ischemic disease concomitant with insulin resistance (63). The NLRP3 inflammasome is a member of the NLR family of innate immune cell sensors. In general, activation of the NLRP3 inflammasome requires two signaling pathways, priming and activating (64). In the priming phase, toll-like receptors (TLRs) recognize a wide variety of danger-associated molecular patterns (65), promote the expression and activation of NLRP1 and NLRP3 inflammasome proteins, and both precursors IL-1β and IL-18 in primary cortical neurons and brain tissue under ischemic conditions. Increased ROS, lysosome rupture, and intracellular potassium efflux activate the NLRP3 inflammasome, promoting the maturation of IL-1β and IL-18 and the release of proinflammatory cytokines (66). Activation of NLRP3 inflammasomes through NF-κB and MAPK signaling pathways to secrete pro-inflammatory cytokines after brain ischemia may be an intrinsic mechanism that enlarges the ischemic damage and causes the disorder of glucose metabolism after stroke (67). The NLRP3 inflammasome may serve as a potential mechanism for the interaction between insulin resistance and ischemic cerebrovascular disease.

### 4.3. Strengths and limitation

To our knowledge, this is the first study to utilize CiteSpace's co-occurrence and co-citation analysis methods for bibliometric analysis and visual display of insulin resistance and ischemic cerebrovascular disease. However, our study does have limitations. Bibliometric studies rely heavily on databases, while our study did not cover other public and commercial bibliometric databases such as PubMed, Scopus, Medline, and CNKI. Therefore, the data may not be comprehensive. But this was caused by the limitations of the software and databases. For example, PubMed does not provide citation analysis. In addition, WOS has the advantages of wide coverage and strong authority, and it can provide better graphics and more detailed content than Scopus in citation analysis (68). Therefore, even if we only analyzed the literature from WOS, the findings remain reliable. Furthermore, some overlap may occur when analyzing the co-occurrence and clustering of keywords due to the presence of multiple synonyms.

## 5. Conclusions

The current study does suggest a strong correlation between insulin resistance and ischemic cerebrovascular disease, but whether it can be an independent influence on poor prognosis after stroke remains controversial. In the future, prospective trials with large samples are needed to deeply analyze the role of insulin resistance in the pathogenesis of ischemic cerebrovascular diseases, such as whether there is a correlation between infarction site, infarction size, and insulin resistance after stroke, what kind of people are more prone to glucose metabolism disorders after stroke, what kind of people with post-stroke glucose metabolism disorder are more likely to have poor clinical outcomes. The IRIS trial demonstrated that pioglitazone can improve the clinical outcome of insulin resistance combined with ischemic cerebrovascular disease, but adverse effects such as bone fractures may limit the clinical use of pioglitazone (69). It may be a new research trend to explore drugs that can improve central insulin resistance, alleviate ischemic injury and promote neurological recovery. At present, SGLT2 inhibitors may serve as the first drug that can improve hypothalamic insulin resistance through the blood-brain barrier (70, 71). Inhibition of the NLRP3 inflammasome activation process and thus inhibition of proinflammatory cytokine release may serve as an early modulable target to improve the inflammatory cascade in patients with insulin resistance combined with ischemic cerebrovascular disease. The intrinsic mechanisms underlying the interaction between insulin resistance and ischemic cerebrovascular disease still need further investigation.

Our research results ultimately provide valuable information for potential collaborators and institutions, teasing out the current status, hotspots, and frontiers of insulin resistance and ischemic cerebrovascular disease, which may guide new directions for further research.

## Author contributions

XZ and CK designed the study, retrieved the data, performed the statistical analysis, and wrote the first draft. YH made further modifications. XW supervised the whole process and provided modification advice. All authors contributed to the article and approved the submitted version.
